# Operative Dentistry

**Published:** 1885-01

**Authors:** Louis Ottofy

**Affiliations:** Ottofy, Dak.


### Operative Dentistry.

BY LOUIS OTTOFY, D.D.S., OTTOFY, DAK.

Under the caption of this paper I wish to treat mainly of the "failures" which are liable to occur in the practice of operative dentistry, with the intention to incite an interesting and profitable discussion of important, undecided questions, rather than to present to you vague ideas as to their cause.

It is generally admitted that one of the most important, if not *the* most important, branch of operative dentistry is that which embraces the preservation of the natural teeth by the means of filling; but in our efforts to preserve the dental organs of all the various persons who are apt to present themselves in a general practice, we are all sometimes baffled, misled, and often finally fail, and this is true not only of us individually, but as a profession, and we might as well stand up as men and acknowledge the fault. The only man whom I ever believed when he told me that no filling of his ever "came out," lived in California, traveled on a mule, and made it a rule never to visit the same place a second time.

These unfavorable, but nevertheless true conditions have led us all more or less to the study of the question: Why do fillings fail? In my opinion, there are numerous answers to this question, most of which are true in their general way. Perhaps, aside from pure carelessness, first, neglecting to properly protect the cervical border of a cavity (where such border exists); second, the matter of selecting the proper material, is the main cause of hundreds of failures. It is certainly a matter of regret that in the selection of filling materials so many outside considerations arise which make it exceedingly difficult to select just the material for the case in hand; so, for instance, how often and how many, who are conscientiously opposed to the general and indiscriminate use of amalgam, are obliged to resort to its use contrary to their wishes? And is it not frequently that gold is used by some over-sanguine but misled dentist, who advocates gold as the *ne plus ultra?* Have there not, in many instances, nasty, black, so-called silver fillings stared you in the face, where circumstances indicate that gold should be? And don't you think many a poor mortal was tortured six or eight hours, with a plugger and a mallet, to find to his sorrow, in a very short time, that human-kind does sometimes err?

It seems a peculiarity of our present infantile position as a profession, that such decidedly different opinions should exist relative to that important question: What is the best material? I have in my mind the name of one gentleman who stands at the head of our profession, and who has frequently made publicly the statement that he has never, nor ever will, put in an amalgam filling; but I can transfer my mind and see as honest, industrious, and true a gentleman, scholar, and successful a practitioner of our profession, as, perhaps, will ever grace the pages of our history, but whose true and honest opinion, so formed by actual experiments, leads him to stand entirely opposite to the foregoing theory as to the proper practice to pursue.

It is my conviction that our failures in filling can be materially decreased, first, by performing all of our operations, whatever the material used, carefully and thoroughly, by that I mean that before a filling is pronounced finished, a conscientious reply of the man to the soul should say that the "best is done;" second, wherever a cervical border to a cavity exists, let it receive twice the care of the previous case; third, use just the material for the case.

As I stated at the beginning of this short paper, my intention was simply to incite a discussion and I therefore now leave to you the open question: What is the best material?

